# Maganese in Chlorosis

**Published:** 1854-12

**Authors:** 


In Chlorosis, the salts of maganese are now very generally substituted for those of iron, in Germany, and the testimony is very strong in their favor, as appears by the foreign journals.—*N. Y. Medical Gazette.*

